# Description of frail older people profiles according to four screening tools applied in primary care settings: a cross sectional analysis

**DOI:** 10.1186/s12877-019-1354-1

**Published:** 2019-12-03

**Authors:** Itziar Vergara, Maider Mateo-Abad, María Carmen Saucedo-Figueredo, Mónica Machón, Alonso Montiel-Luque, Kalliopi Vrotsou, María Antonia Nava del Val, Ana Díez-Ruiz, Carolina Güell, Ander Matheu, Antonio Bueno, Jazmina Núñez, Francisco Rivas-Ruiz

**Affiliations:** 1 Unidad de Investigación APOSIs Gipuzkoa, Osakidetza, Instituto de Investigación Sanitaria Biodonostia, 20014 Donostia-San Sebastian, Spain; 2 Instituto Biodonostia, Donostia-San Sebastian, Spain; 3 Red de Investigación en Servicios de Salud en Enfermedades Crónicas (REDISSEC), Madrid, Spain; 4 Kronikgune, Barakaldo, Spain; 5 Unidad Gestión Clínica Los Boliches, Fuengirola, Distrito de Atención Primaria Costa del Sol, Málaga, Spain; 6 Unidad Gestión Clínica San Miguel, Torremolinos. Distrito de Atención Primaria Costa del Sol, Málaga, Spain; 7 Unidad Gestión Clínica Las Albarizas, Marbella, Distrito de Atención Primaria Costa del Sol, Málaga, Spain; 8 Centro de Salud Beraun, OSI Donostialdea, Osakidetza, Errenteria, Spain; 9 Grupo de Oncología Celular, Instituto Biodonostia, San Sebastián, Spain; IKERBASQUE, Fundación Vasca para la Ciencia, Bilbao, Spain; CIBER de Fragilidad y Envejecimiento Saludable (CIBERFES), Bilbao, Spain; 10 Unidad de Investigación Agencia Sanitaria Costa del Sol, Marbella, Málaga, Spain

**Keywords:** Frailty, Community, Screening, Primary care

## Abstract

**Background:**

Regarding the health care of older populations, WHO recommends shifting from disease-driven attention models towards a personalized, integrated and continuous care aimed to the maintenance and enhancement of functional capacities. Impairments in the construct of functional intrinsic capacity have been understood as the condition of frailty or vulnerability. No consensus has been yet reached regarding which tools are the most suitable for screening this kind of patients in primary care settings. Tools based on the measurement of functional performance such as Timed up and go test (TUG), Short Physical Performance battery (SPPB), self-completed questionnaires like Tilburg Frailty Indicator (TFI) and clinical judgement, as the Gerontopole Frailty Scale (GFS) may be adequate. The objective of this work is to describe and compare characteristics of community-dwelling individuals identified as vulnerable or frail by four tools applied in primary care settings.

**Methods:**

Cross sectional analysis developed in primary care services in two regions of Spain.

Community-dwelling independent individuals aged 70 or more willing to participate were recruited and data was collected via face-to-face interviews. Frailty was assessed by TUG, SPPB, TFI and GFST. Also socio-demographic characteristics, lifestyle habits and health status data (comorbidities, polypharmacy, self-perceived health), were collected. Multiple correspondence analysis (MCA) and cluster analysis were used to identify groups of individuals with similar characteristics.

**Results:**

Eight hundred sixty-five individuals were recruited, 53% women, with a mean age of 78 years. Four clusters of participants emerge. Cluster 1 (*N* = 263) contained patients categorized as robust by most of the studied tools, whereas clusters 2 (*N* = 199), 3 (*N* = 183) and 4 (*N* = 220) grouped patients classified as frail or vulnerable by at least one of the tools. Significant differences were found between clusters.

**Conclusions:**

The assessed tools identify different profiles of patients according to their theoretical construct of frailty. There is a group of patients that are identified by TUG and SPPB but not by GFS or TFI. These tools may be useful in primary care settings for the implementation of a function- driven clinical care of older patients.

## Background

The World Report on Ageing and Health published by the World Health Organization (WHO) in 2014 [1] provides a conceptual framework for a new approach to the health care of older populations. It shifts from a disease-driven attention towards a healthy ageing idea [2]; the latter being characterized by a personalized, integrated and continuous care aimed at the maintenance and enhancement of functional capacities regardless of clinical phenotypes.

The key concept of this framework is functional capacity. As defined in the above mentioned document, “Functional capacity comprises the attributes that enable people to do what they have reason to value” and it is made up of two components: the intrinsic capacity and the environment [1]. Impairments in the construct of intrinsic capacity have been understood as the condition of frailty [3]. One consensus definition describes ‘frailty as a medical syndrome with multiple causes and contributors that is characterized by diminished strength, endurance, and reduced physiologic function that increases an individual’s vulnerability for developing increased dependency and/ or death’ [4]. The two most widely accepted models that conceptualise frailty are Fried’s Phenotype [5] and the Cumulative deficit Model of the Canadian Study of Health and Aging (CSHA) [6].

Based on these models a huge number of tools have been proposed to screen and diagnose frailty in clinical settings. To date, more than eight systematic reviews in addition to numerous other articles have been published analysing the performance of different instruments for the screening or the assessment of frailty [7–16]. These tools are based on diverse approaches: some of them on multicomponent assessments [17–21], while others are single outcome oriented [22–24]. Their administration also differs: some are based on clinical record information [18, 25, 26]; some are self-completed or auto-reported [27–30]; and some others depend on professional assessment and clinical judgement [31, 32].

At the primary care level, adequate simple tools are needed for frail patients to be identified [33]. In the last years, a number of tools has been specifically developed and validated to some extent in primary care settings [13, 32, 34]. To date, they haven’t been incorporated into routine practice [35].

With the debate about the appropriateness and need for frailty screening and identification in primary care widely open [36], complementary information is needed to define the most informative tool to be used in this specific clinical setting. It is relevant to consider that different tools provide distinct and complementary clinical information about the risk profile of an older person and that to preserve functional capacity early actions in persons presenting increased risk profile are needed [37]. This is why we aimed to describe the characteristics of community-dwelling frail individuals identified as vulnerable or frail by four tools in order to understand what profile of patients was being identified by each tool. That could help to provide new insights about the performance of these tools when applied to primary care settings and in the selection of the most adequate tool for this specific clinical setting to implement the recommended functional capacity-driven care model.

## Methods

The analyses reported here are based on data obtained at the baseline assessment (May 2015 to July 2016) of a multicentre prospective cohort study with 2 years of follow-up, which methodology has been described elsewhere [38]. The study was conducted in two regions of Spain, the Basque Country and Andalusia located in the north and south shores, respectively. Participants were included according to the following inclusion criteria: community-dwelling, functionally independent (Barthel Index >90 points), aged 70 or more and provision of informed consent. Only non-dependent patients were included, as the occurrence of dependence was one of the health adverse outcomes that were going to be measured in the cohort study. At baseline, data were collected via face-to-face interviews by trained nurses on the following variables: frailty, socio-demographic characteristics, lifestyle habits and health status (comorbidities, polypharmacy, self-perceived health), among others. Taking in consideration the clinical practice characteristics in primary care settings, tools based on the measurement of clinical performance, self-completed questionnaires and clinical judgement for the identification of frail vulnerable patients seem promising. This is why, for the purpose of this study the Timed Up and Go test (TUG), the Short Physical Performance Battery (SPPB), the Tilburg Frailty Indicator (TFI) and the Gérontopôle Frailty Screening Tool (GFST) were chosen.

The TUG measures the time an adult needs to get up from a chair, walk 3 m, turn around, come back to the chair and sit down again. Depending on the time needed to do the above tasks, subjects are categorized as frail or robust [39]. Different cut off points have been proposed but for the purpose of this study subjects with performance times higher than 12 s [40] were considered frail. The SPPB includes three objective tests of lower body function [41]. A summary score was created with a potential range of 0–12, with a total score < 10 considered indicative of frailty [24, 42, 43]. TFI is a 15-item self-administered questionnaire related to 3 domains: physical, psychological and social. Its total score ranges from 0 to 15 points. Scores ≥5 indicate frailty [28]. An assessment of the psychometric properties of the Spanish TFI adaptation is described elsewhere [44].

The GFST is administered by physicians to non-dependent older patients without current acute disease. Based on an initial questionnaire aimed at attracting the general practitioner’s attention to very general signs and/or symptoms suggesting the presence of an underlying frailty status, the health care professional is asked whether in his/her clinical opinion the patient is frail or robust [31]. Participants were assessed by trained health care professionals using all of these four tools during a single interview session.

### Statistical analysis

Categorical variables are presented as frequencies and percentages, n (%), and continuous variables as mean and standard deviation (SD) when normally distributed and otherwise as median and quartiles 1 and 3 (Q1, Q3). Comparisons between groups were carried out using the chi-square test for categorical variables and Student’s t-test or the non-parametric Wilcoxon rank-sum test for continuous variables.

Multiple correspondence analysis (MCA) and cluster analysis were used to summarize the information obtained by the four tools and to analyse groups of individuals. MCA is a technique that summarizes information into a few components which explain the maximum amount of variability contained in the active variables included in the analysis. This multivariate technique is a useful tool to determine the relationship between categorical variables and has been widely used in medical research [45, 46].

First, we performed the MCA including all participants and variables from the tools used to categorize them as robust or frail as active variables. In addition, we included sex as an illustrative variable. The results are interpreted using graphs based on the components of the MCA. Categories of the variables included in the analysis are displayed in a two-dimensional map, on which the variables and individuals coordinates are represented for each component: the closer the points, the stronger the association.

Second, a hierarchical cluster analysis was used to organize all participants into groups of similar individuals. Component coordinates provided by the MCA were used to measure differences and define groups of individuals.

Finally, the resulting groups were characterized and the individuals were plotted on the MCA map, in order to visualize each group. Groups that emerged from this analysis were compared.

All the analyses were performed using the free statistical software R, version 3.4.0.

## Results

Patients who initially met the inclusion criteria, according to their health clinical record information, were contacted and invited to participate (*N* = 2420). A total of *N* = 885 accepted participation, with *N* = 865 finally fulfilling the study inclusion criteria. Presented results are based on the latter sample. The overall mean age was 78.2 (SD: 4.9) years and 53% were women (Table 1). Participants had a low educational and income level. Most subjects were non-smokers (94%) and 37% were obese. They presented a high degree of comorbidity, with an age-adjusted Charlson Index of 4.5 (SD: 1.4), the most frequent diseases being diabetes mellitus (44%; 6% with organ affection), COPD (21%) and congestive heart failure (18%) (data not shown). Besides, 19% of the participants had hearing problems and 15% had visual impairment, while 30% had a fall during the previous year. The four studied tools yielded different prevalence rates of frailty: 38% (95%CI 35–41%), 55% (95%CI 52–59%), 29% (95%CI 26–32%) and 31% (95%CI 28–34%) for the TUG, SPPB, TFI and GFST, respectively. In all tools except for the GFST, significant differences were observed by sex, the prevalence being higher in women.

**Table 1:** Baseline characteristics of the participants

	Total	missing
N	865	
Age, years; mean (SD)	78.2 (4.9)	4
Sex (female)	458 (53)	0
Education level		14
Primary	689 (81)	
Secondary	56 (7)	
Higher	106 (12)	
Income (≤€1200)	508 (62)	41
Tobacco consumption (non-smoker)	807 (94)	3
Body mass index >30 kg/m^2^	321 (37)	1
Low physical activity level	111 (13)	7
Visual impairments	130 (15)	1
Hearing impairments	167 (19)	1
Falls during the last year	256 (30)	3
Age-adjusted CCI; mean (SD)	4.5 (1.4)	4
Self-perceived health status		0
Good	634 (73)	
Poor	231 (27)	
Number of drugs; median (Q1, Q3)	5 (3,7)	1
Polypharmacy	595 (69)	1

Data presented as frequencies (percentages), n (%), otherwise stated; *N* number of observations; *CCI* Charlson Comorbidity Index

**Fig. 1:**
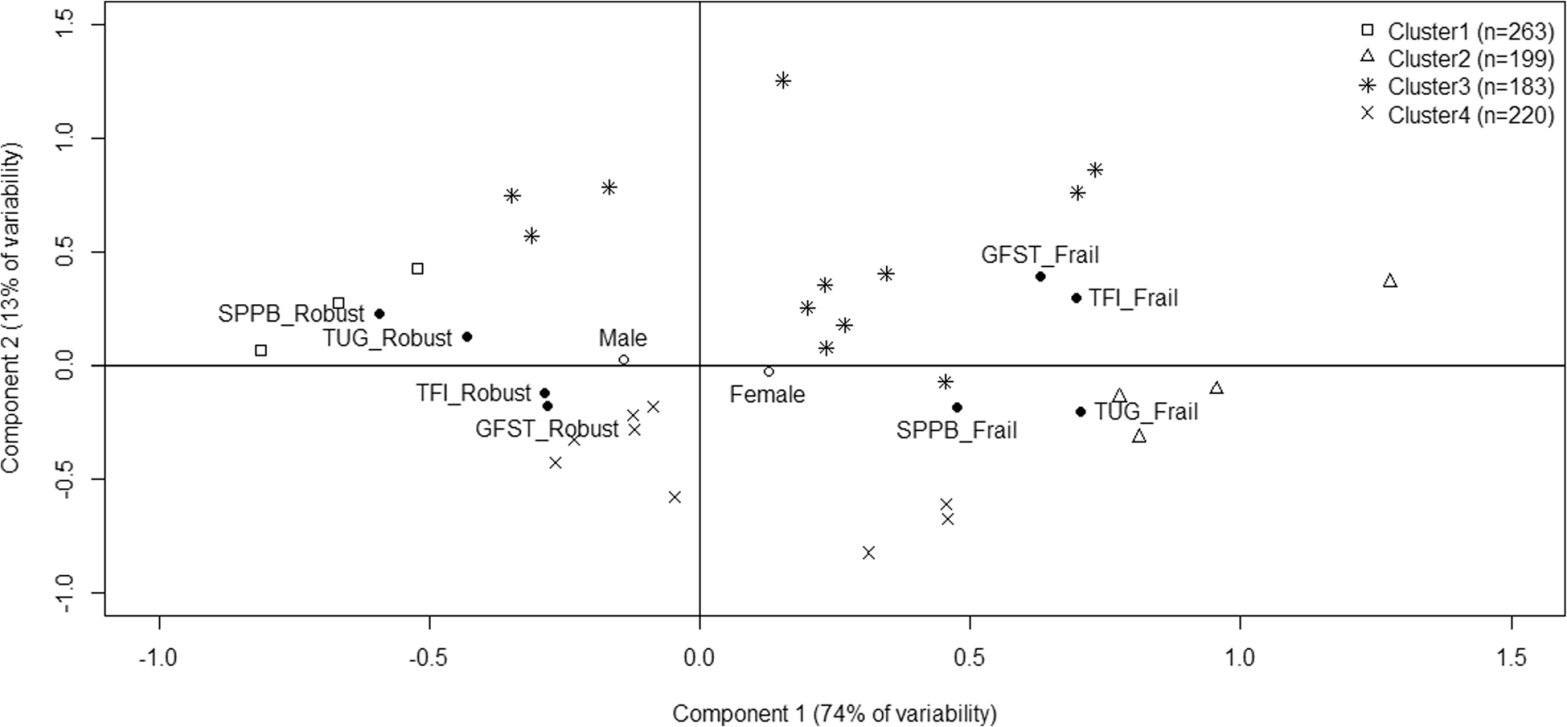
Graphical display of the four clusters in the first two components of the multiple correspondence analysis. TUG, Timed Up and Go Test; SPPB, Short Physical Performance Battery; TFI, Tilburg Frailty Indicator; GFST, Gérontopôle Frailty Screening Tool. The first component can be interpreted as robust (left side) vs frail (right side) index. The second component seems to differentiate two types of frailty: functional frailty as measured by the SPPB or TUG (bottom) and frailty identified by clinical judgment or self-report of health and social status as measured by the GFST or TFI (top). Black dots in the plane represent the categories of the active variables included in the multiple correspondence analysis, empty dots represent the sex, included as illustrative variable in the analysis. The closer the points are, the stronger the relationship between the categories. Relative positions of the subjects in this plane are represented by different symbols, depending on the subtype provided by the cluster analysis

The results of the multiple correspondence analyses and the cluster analysis are shown in Fig. 1. Two main components explained 74 and 13% of the variance, respectively. The first component distinguished between robust (left side of the figure) and frail (right side of the figure) individuals. The second component seemed to differentiate two types of frailty: one that could be defined as functional frailty, as measured by the SPPB or TUG, (bottom of the figure) and the other identified by clinical judgment or self-report of the individual’s health status, as measured by GFST or TFI (top of the figure). Considering these components, four clusters of participants emerge. Cluster 1 (*N* = 263) contained patients categorized as robust by all four tools, whereas clusters 2 (*N* = 199), 3 (*N* = 183) and 4 (*N* = 220) grouped patients classified as frail by at least one of the tools (Table 2).

**Table 2:** Characterization of the cluster of individuals and comparison between frail clusters

	Cluster 1^a^	Cluster 2^b^	Cluster 3^b^	Cluster 4^b^	p-value^c^
N	263	199	183	220	
Age, years; mean (SD)	77.3 (4.6)	78.9 (5.8)	78.4 (4.7)	78.2 (4.5)	0.314
Sex (female)	110 (42)	137 (69)	87 (47)	124 (56)	<0.001
Income (≤€1200)	132 (53)	140 (74)	109 (61)	127 (62)	0.012
Body mass index >30 kg/m^2^	84 (32)	90 (45)	56 (31)	91 (42)	0.010
Low physical activity level	8 (3)	63 (32)	22 (12)	18 (8)	<0.001
Visual impairments	19 (7)	47 (24)	36 (20)	28 (13)	0.015
Hearing impairments	37 (14)	50 (25)	42 (23)	38 (17)	0.136
Falls in the last year	60 (23)	85 (43)	61 (33)	50 (23)	<0.001
Age-adjusted CCI; mean (SD)	4.1 (1.2)	4.9 (1.4)	4.8 (1.6)	4.3 (1.3)	<0.001
Self-perceived health status					<0.001
Good	240 (91)	90 (45)	126 (69)	178 (81)	
Poor	23 (9)	109 (55)	57 (31)	42 (19)	
Number of drugs; median (Q1, Q3)	4 (2,6)	7 (5,9)	6 (4,8)	5 (3,6)	<0.001
Polypharmacy (≥4 drugs)	146 (55)	170 (86)	141 (77)	138 (63)	<0.001
Frailty					
TUG (Frail)	0 (0)	199 (100)	21 (11)	108 (49)	<0.001
SPPB (Frail)	0 (0)	199 (100)	78 (43)	203 (92)	<0.001
TFI (Frail)	0 (0)	138 (69)	110 (61)	0 (0)	<0.001
GFST (Frail)	0 (0)	143 (73)	119 (65)	0 (0)	<0.001

Data are presented as frequencies (percentages), n (%), otherwise stated; *N* = number of observations; *CCI* Charlson Comorbidity Index, *TUG* Timed Up and Go Test, *SPPB* Short Physical Performance Battery, *TFI* Tilburg Frailty Indicator, *GFST* Gérontopôle Frailty Screening Tool

^a^Cluster 1 = patients categorized as robust by all four studied tools

^b^Clusters 2, 3 and 4 = patients classified as frail by at least one of the tools

^c^*p*-values = based on comparisons between Clusters 2, 3 and 4

All variables shown in the table were found to be statistically significant (*p* < 0.05) when comparing robust (Cluster 1) versus frail groups (Clusters 2, 3 and 4)

Significant differences were found between clusters (Table 2). In particular, notable differences were observed between robust (cluster 1) and frail (cluster 2, 3 and 4) patients, as expected. Robust patients were younger (77.3 years [SD: 4.6]), with a higher level of physical activity (only 3% low level) and lower rates of hearing (14%) and sight problems (7%); they were less likely to have a history of falls (23%), and were more often male (58%). The level of comorbidity was also lower (80% having an age-adjusted Charlson Index of 0 or 1, data not shown), and took fewer prescription drugs than those in the frail clusters (*p* < 0.001). They also had a better self-perceived health status with 91% rating their health as good.

Additionally, relevant differences could be found between clusters 2, 3 and 4, enabling to identify different profiles of frail patients. Cluster 2 gathered patients identified as frail by, at least three of the tools: TUG (100%) and SPPB (100%), TFI (69%), GFST (73%). They were more likely to be women (69%), have a history of falls (43%), and have high levels of comorbidity (age-adjusted Charlson Index 4.9, SD: 1.4) and polypharmacy (median:7; Q1, Q3: 5,9), high rates of hearing (25%) and visual (24%) problems, low levels of income (74% having an income of <€1200/month) and of physical activity (32%), and poor self-perceived health status (55% rating their health as poor).

Cluster 3 is constituted by patients mostly identified as frail by the TFI (61%) or GFST (65%) and, to a lesser extent, by the SPPB (43%) or TUG (11%). These patients were mostly similar to cluster 2 regarding the levels of comorbidity (age-adjusted Charlson Index 4.8, SD: 1.6) and polypharmacy (median: 6; Q1, Q3: 4, 8), but were slightly less likely to have a history of falls in the last year (33%), and hearing (23%) or sight problems (20%). Further, this cluster had a better self-perceived health (69% rating their health as good) and a lower percentage of women (47%) than cluster 2.

Finally, cluster 4, contained individuals identified as frail by the TUG (49%) and SPPB (92%) and none classified as frail by the TFI or GFST. This cluster was balanced regarding sex (with a slightly higher percentage of women, 56.4%) and, compared to others had a higher level of physical activity (low level 8%), but still a relatively high percentage had a history of falls (23%). The greatest differences were found in the level of comorbidity, with most patients (78%) having no comorbidities at all (data not shown), a lower prevalence of polypharmacy (37% not taking multiple medications) and the high frequency of good self-perceived health (81%).

## Discussion

To the best of our knowledge, this is one of the few studies that, in addition to comparing different tools for assessing frailty, go in depth in the description of the individuals classified by these tools using Multiple Correspondence Analysis and cluster analyses [47–51]. It is relevant to note that the tools implemented in this work were selected after considering the available instruments at the time the current study was proposed and approved. The four studied tools represent different approaches to the identification of frail individuals that were both feasible and informative for primary care settings. The TFI was considered because it appeared to be potentially relevant for the screening of frailty in primary care [52] and because its method of data collection is easy to use in primary care. Besides, it is also worth mentioning that this group has translated and culturally adapted the TFI for use in Spain [44]. The GFST was included, even though it was not validated at the time, because it was based on clinical judgement and this was a relevant approach for primary care settings in our opinion. Later, other tools based on clinical judgement were described and validated [53]. Functional performance tests of TUG and SPPB were included because they have been proposed as tools for the identification of frail individuals [23, 24] and also because they are recommended in the algorithm for the identification of frail patients by the Spanish Ministry of Health [43]. The phenotype proposed by Fried et al. has not been considered in this study given its difficulties to be applied in the clinical setting of interest [12, 36, 54].

Regarding our findings, when these four tools are used simultaneously a key issue emerges: the different characteristics of those identified as frail or vulnerable by each tool. The difference between profiles is clearly explained by the differences among the underlying theoretical approaches of the explored tools. The TUG and SPPB rely on the measurement of the capacity to perform physical tasks based on muscle mass and coordination mainly of the lower body. On the other hand, the TFI explores other aspects of frailty related to self-perceived health and social support, and the GFST is based on clinical judgement and the impression of severity.

The differences observed between robust (cluster 1) and all frail patients (clusters 2, 3 and 4) are already known and consistent with the construct of frailty. The differences observed between the three clusters that grouped frail patients are more interesting. Patients in cluster 2 are identified as frail by most of the studied tools. They have a high level of comorbidity, a low level of functional performance, poor self-perceived health and a low income, and hence, health-related adverse outcomes could be expected. The comparison between clusters 3 and 4, however, is more revealing. Cluster 3 corresponds to individuals with a high degree of comorbidity and polypharmacy who are identified as frail by the TFI and GFST, whereas cluster 4 patients have relatively few health problems but notably impaired functional performance as identified by the TUG and SPPB. It is important to highlight that none of these patients in cluster 4 are identified as frail by TFI or GFST. Physicians did not diagnose frailty according to the GFST, neither the patients see themselves as vulnerable or frail according to the TFI; nonetheless they actually do have a high risk of adverse effects considering the proven predictive capacity of TUG and SPPB for such events [39, 54].

These results provide evidence that the TUG and SPPB tools identify a set of patients not identified by the other studied tools [39, 55]. There is some controversy regarding the effectiveness of interventions aimed to reduce the level of frailty or to reduce the incidence of adverse effects related to it [56, 57]. But, there is a sound consensus on the need to tackle the health needs that may jeopardize aged patients’ functional capacity. Overall, these results provide evidence on the relevance of the decision about which tools are the most informative to be used in primary care where frail and vulnerable patients need to be identified [49].

The main limitation of this study is related to the representativeness of the sample given the natural tendency of individuals with better heath to be more likely to participate. It is important to be aware that this study is based on the cross sectional analysis of the baseline data of a follow up study, so only descriptive results are provided. Also it has to be noted that the selection of the studied tools was made considering the evidence available at the time the study was designed and conducted. The approach used in this study, combining multivariate techniques with cluster analysis, is a notable strength. These techniques and their combination are used to differentiate groups of individuals and to describe them in the context of the groups formed [47].

One of the ways to implement the functional capacity-driven care for aged patients is to identify those at risk of losing it in order to activate early actions to contain and decrease that risk. Primary care professionals should be more involved in the care for functional capacity through the identification of vulnerable and frail people and should also recognize their role in tackling age related conditions promoting primary preventive actions in the community in collaboration with public healthcare authorities [38] .

## Conclusions

Thoughtful reflection is required to clarify what kind of frail and vulnerable individuals would benefit from being identified and selected for management in primary care: those who are very sick and are already known to their health professionals or those that are losing their functional abilities, becoming weak and silently losing speed and balance.

More longitudinal research, and clear clinical targets and endpoints are needed to assess the effectiveness of interventions targeting these patients in order to provide a sound answer to this question. Until more evidence is available, according to our results, TUG and SPPB may be useful for the identification of a group of patients that are not identified by other tools and that may benefit from interventions that improve their functional capacity in primary care settings.

## Data Availability

The datasets used and analyzed during the current study are available from the corresponding author on reasonable request.
